# Evaluation of Sample Preparation Strategies for Human Milk and Plasma Proteomics

**DOI:** 10.3390/molecules26226816

**Published:** 2021-11-11

**Authors:** Sanja Milkovska-Stamenova, Michele Wölk, Ralf Hoffmann

**Affiliations:** 1 Institute of Bioanalytical Chemistry, Faculty of Chemistry and Mineralogy, Universität Leipzig, Deutscher Platz 5, 04103 Leipzig, Germany; michele.woelk@uni-leipzig.de (M.W.); bioanaly@rz.uni-leipzig.de (R.H.); 2 Center for Biotechnology and Biomedicine, Universität Leipzig, Deutscher Platz 5, 04103 Leipzig, Germany

**Keywords:** bottom-up proteomics, filter-aided sample preparation (FASP), Folch extraction, in-solution digestion (ISD), milk, plasma

## Abstract

Sample preparation is the most critical step in proteomics as it directly affects the subset of proteins and peptides that can be reliably identified and quantified. Although a variety of efficient and reproducible sample preparation strategies have been developed, their applicability and efficacy depends much on the biological sample. Here, three approaches were evaluated for the human milk and plasma proteomes. Protein extracts were digested either in an ultrafiltration unit (filter-aided sample preparation, FASP) or in-solution (ISD). ISD samples were desalted by solid-phase extraction prior to nRPC-ESI-MS/MS. Additionally, milk and plasma samples were directly digested by FASP without prior protein precipitation. Each strategy provided inherent advantages and disadvantages for milk and plasma. FASP appeared to be the most time efficient procedure with a low miscleavage rate when used for a biological sample aliquot, but quantitation was less reproducible. A prior protein precipitation step improved the quantitation by FASP due to significantly higher peak areas for plasma and a much better reproducibility for milk. Moreover, the miscleavage rate for milk, the identification rate for plasma, and the carbamidomethylation efficiency were improved. In contrast, ISD of both milk and plasma resulted in higher miscleavage rates and is therefore less suitable for targeted proteomics.

## 1. Introduction

Mass spectrometry (MS-) based proteomics is a powerful tool to identify and quantify proteins as well as to study protein–protein interactions in various biological matrices. It relies on several critical key steps from sample preparation via MS to data analysis with each being crucial to obtain reliable and reproducible results. In a typical bottom-up proteomics workflow, proteins are extracted from the sample, reconstituted, reduced, alkylated, cleaved with a protease like trypsin, desalted, and analyzed by liquid chromatography (LC-) MS [1]. Some protocols using substances incompatible with consecutive digestion and LC-MS, such as detergents and chaotropic salts facilitating protein solubilization, require additional procedures to remove these interfering substances [2]. Furthermore, biological fluids, such as human milk and plasma, contain many substances at high concentrations, such as sugars and lipids, which can interfere with LC separation and the electrospray ionization (ESI) process suppressing signals in MS. Thus, it is important to remove these substances from protein samples during initial sample preparation, classically by protein precipitation or ultrafiltration in order to avoid artefacts [3,4,5]. This is especially important when studying nonenzymatic posttranslational modifications that can be easily induced during sample preparation, for example, reducing sugars can react with amino groups in proteins (protein glycation) [6]. Folch extraction using methanol, chloroform, and water separates lipids (organic phase) from sugars and other polar small substances (aqueous phase), while proteins precipitate in a layer between both phases. This procedure reproducibly yields higher protein recoveries (~90%) for bovine milk than other commonly used precipitation procedures using trichloroacetic acid (TCA) or acetone and MTBE extraction [7].

Among the commonly used digestion strategies, the in-solution digestion (ISD) is the simplest and most powerful approach, because denaturation, reduction, alkylation, and digestion are performed in a single tube minimizing potential sample losses. As reagents added during ISD may interfere with LC-MS, solid phase extraction (SPE) is typically used to purify and concentrate peptides [8]. Alternatively, ISD can be performed in an ultrafiltration unit, so-called filter-aided sample preparation (FASP), published in 2005 [9]. In this approach, proteins are trapped in an ultrafiltration unit with a high molecular weight cutoff (MWCO), whereas small molecules can be discarded [10]. Thus, FASP directly removes detergents and contaminants from samples and does not require a subsequent clean up procedure.

None of these sample preparation methods performs best for all sample types. As an ideal universal sample preparation is missing, it is necessary to identify for each type of biological sample the best procedure allowing a reliable identification and quantitation of peptides and proteins. Consequently, this study compared sample preparation procedures for complex sample matrices, i.e., human milk and human plasma, considering protein precipitation and ultrafiltration to remove small molecules including detergents and to isolate proteins followed by tryptic digestion performed in-solution and by FASP. The methods were compared in terms of protein and peptide identifications, efficiency of digestion (number of missed cleavage sites), quantitation, and reproducibility. All three procedures allowed a reproducible profiling and quantitation of milk and plasma proteins. FASP yielded less missed cleavage sites and is, therefore, preferred for quantifying targeted peptides. Protein precipitation prior to FASP seems to be beneficial due to less miscleavages of milk proteins and a better carbamidomethylation rate in plasma. In quantitative terms, protein precipitation followed by FASP provided higher signals than ISD for plasma and a significantly better reproducibility for milk.

## 2. Results

### 2.1. Identification of DnaK Peptides

DnaK recombinantly expressed in *E. coli* was digested in triplicates using FASP or ISD, and these were analyzed by nRPC-ESI-MS/MS. Considering an FDR of 1%, PEAKS proposed 91 tryptic peptides of DnaK corresponding to a sequence coverage of 91% (Table S1). About 55% of these peptides were identified by both methods, whereas 14 and 26 peptides were only identified in the FASP and ISD samples, respectively (Figure S1). Although, fewer peptides were identified by FASP, this method provided a better sequence coverage of 87% (64 peptides) than ISD covering only 70% of the DnaK sequence (76 peptides). The lower sequence coverage despite more identified peptides by ISD was related to a high number of peptides with one or two missed cleavage sites (Figure S1B,C). Only 40% of the peptides identified in the FASP samples contained at least one missed cleavage site, whereas in ISD about 42% and 17% of the identified peptides contained one and two missed cleavage sites, respectively. The missed cleavage sites produced by FASP were mainly located at the termini of the peptides, whereas ISD produced many longer peptides with missed cleavage sites in mid-chain positions.

### 2.2. Relative Quantitation of DnaK Peptides

After validation in Skyline and excluding peptides with poor MS/MS quality, DnaK peptides with S/N ≥10 were relatively quantified by the peak areas between FASP and ISD. About 60% of the peptides showed different peak areas between both methods. Most peptides without missed cleavages sites were more intense in FASP, whereas peptides with one or two missed cleavage sites were more dominant in ISD (Figure S2). FASP occasionally produced a higher peak area for peptides with one missed cleavage site (Figure S2, panels E–F), but peptides with two missed cleavage sites were not quantifiable in FASP. Interestingly, the sum of all peak areas of quantified peptides was similar between both approaches (Figure S3), but the relative standard deviation was lower for ISD.

### 2.3. Human Milk and Plasma

**Figure 1:**
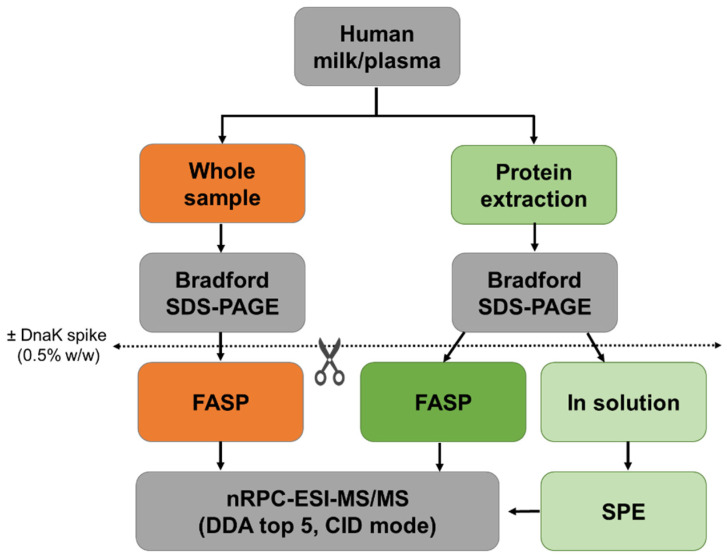
Schematic representation of the workflow. Protein concentrations of human milk and plasma were determined before (orange) and after protein extraction (green). Whole and extracted samples were digested by FASP, and extracted samples were also digested in-solution. Experiments were performed in triplicates with and without the addition of DnaK as an internal control protein.

Different sample preparation strategies and the advantage of a spiked control protein were tested on human milk and plasma representing complex protein mixtures containing diverse non-proteinogenic substances at partially high concentrations (Figure 1). Overall six replicates per sample type, three times with or without spiked DnaK, were analyzed by nRPC-ESI-MS/MS for each strategy. The database searches using PEAKS (1% FDR) proposed in total 1664 peptides from 325 milk protein groups and 2405 peptides from 551 plasma protein groups (Tables S2 and S3, respectively). The term protein group is used here as defined by PEAKS as “proteins identified by the same set of peptides”. As the numbers of peptides identified in individual samples did not depend on spiked DnaK (data not shown), the data presented below referred to spiked samples. It should be noted that this study did not aim for an in-depth characterization of human milk and plasma proteomes.

**Figure 2:**
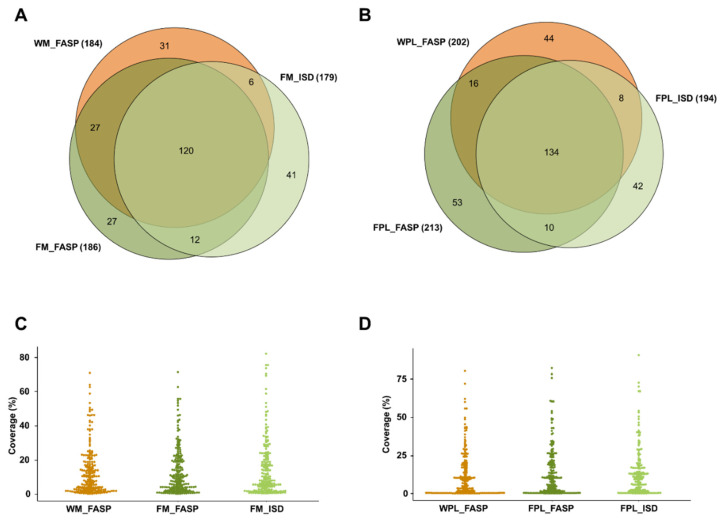
Quantitative Venn diagrams for proteins (**A**) identified in human milk (M) and (**B**) plasma (PL) identified after digesting a diluted aliquot of the whole sample (W) by FASP as well as protein extracts (F) by FASP and in-solution digestion (ISD). Percentage protein coverages for (**C**) human milk and (**D**) plasma grouped by the sample preparation method. Experiments were performed in three independent replicates. Identifications are presented as total numbers of unique proteins for each procedure.

Overall, 264 milk (307 plasma) protein groups were found in samples containing the DnaK spike with ~62% (54.7%) of the proteins being identified by at least two of the applied three methods (Figure 2). The protein coverage was typically below 25%, but in some cases the sequence coverage was higher independent of the applied method (Figure 2C,D). 

**Figure 3:**
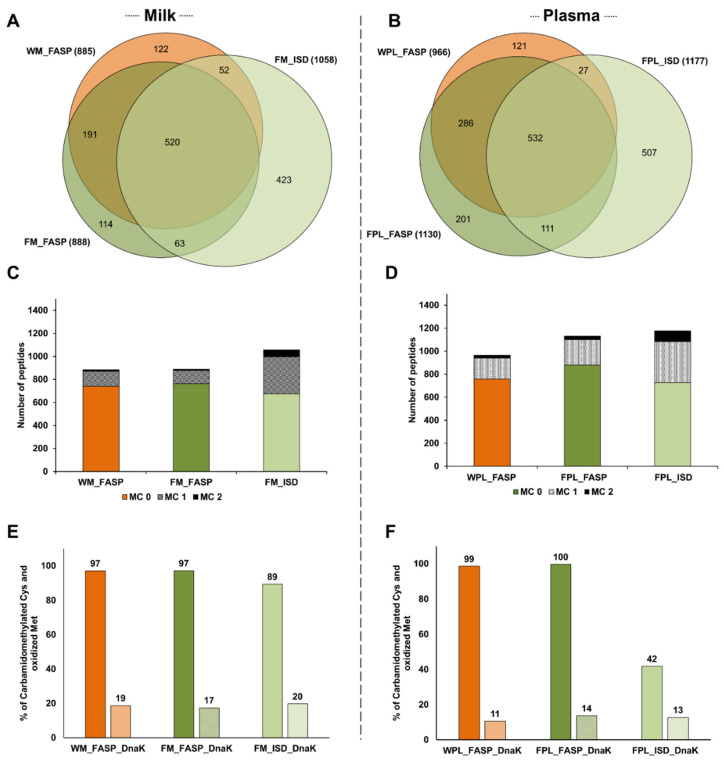
Quantitative Venn diagrams for the number of peptides identified in (**A**) human milk (M) and (**B**) plasma (PL) after digesting a diluted aliquot of the whole sample (W) by FASP as well as protein extracts (F) by FASP and in-solution digestion (ISD). The numbers of peptides with up to two missed cleavages (MC) identified in (**C**) human milk and (**D**) plasma. Efficiency of carbamidomethylation and methionine oxidation as % observed for cysteine/methionine containing sequences in (**E**) human milk and (**F**) plasma. Experiments were performed in three independent replicates. Identifications are presented as total numbers of unique peptides for each procedure.

Considering the 1485 peptides identified only in milk spiked with DnaK, 1058 peptides were detected after Folch precipitation and ISD (FM_ISD), 888 peptides after Folch precipitation and FASP (FM_FASP), and 885 after FASP without prior precipitation (WM_FASP) with ~35% of the peptides identified by all three strategies (Figure 3A). The overlap was higher between the two FASP procedures (~67%). In plasma, the lowest number of peptides was observed after FASP without prior precipitation (WPL_FASP, 966 peptides), while similar numbers of peptides were identified by ISD (1177 peptides) and FASP (1130 peptides) after protein precipitation (Figure 3B). From 1785 peptides identified in the different plasma digests altogether, ~30% were identified by all three strategies and 64% were identified after FASP. Again, many peptides were solely identified in the ISD sample.

The higher number of peptides identified in the ISD samples of milk and plasma resulted from missed cleavage sites leading to additional longer peptides (Figure 3C,D). In milk, only ~14.5% and ~16.4% of all peptides identified in the FM_FASP and WM_FASP samples, respectively, contained one or two missed cleavage sites compared to ~36.2% in FM_ISD (Figure S4A). Similarly, in the plasma samples less than 25% of the peptides detected by either FASP strategy contained at least one missed cleavage site, but ~38.2% in FPL_ISD (Figure S4B). Thus, ISD produced more peptides with one and especially with two missed cleavage sites than FASP (Figure 3C,D), which did not improve the sequence coverage significantly.

The efficiency of reduction and alkylation was judged by the number of Cys(CM) residues (+57.021 Da). Oxidation during sample processing was judged by the number of Met(Ox) residues (+15.99 Da). Therefore, Cys- and Met-containing sequences were counted and the percentage of modified residues calculated. All three protocols provided a high degree of carbamidomethylation in milk samples, with a slightly lower level in the ISD sample, and similar degrees of Met(Ox) (Figure 3E). In plasma, all Cys-containing peptides were carbamidomethylated after precipitation and FASP, whereas only 42% were alkylated after ISD (Figure 3F). Again, Met(Ox) was observed at similar degrees in all three conditions (Figure 3F). 

#### Quantitation

**Figure 4:**
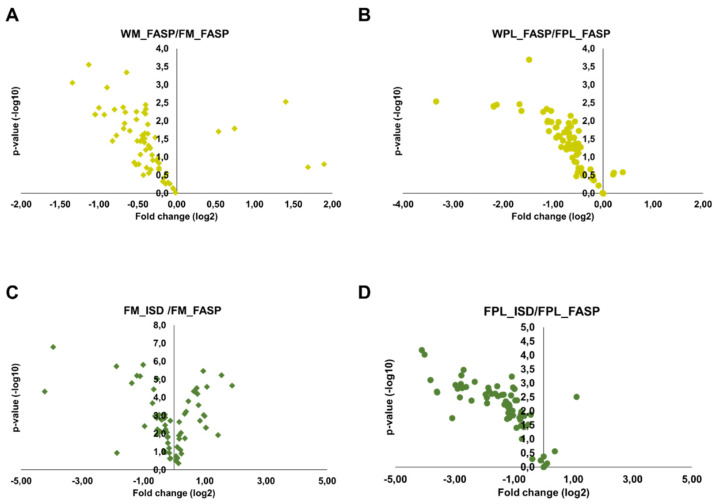
Volcano plots displaying fold changes in protein abundances between the different digestion strategies and the statistical value. The fold change was calculated by dividing the whole sample (FASP digestion) by the Folch precipitated sample (FASP digestion) for (**A**) milk and (**B**) plasma and by dividing the Folch precipitated sample (in-solution digestion) by the Folch precipitated sample (FASP digestion) for (**C**) milk and (**D**) plasma. Experiments were performed in three independent replicates.

Only peptides validated in Skyline and with a signal-to-noise ratio of at least 10 were quantified using the peak areas in XICs. As described above for DnaK, many peptides with missed cleavage sites were observed in the ISD samples. Peptides with no missed cleavage sites showed different, typically protein dependent trends. Importantly, FASP after precipitation of both milk and plasma proteins typically yielded higher peak areas of quantifiable peptides than FASP alone, which might be attributed to a more efficient digestion, higher peptide recovery rates or fewer contaminations suppressing the peptide signals despite the likely loss of proteins during precipitation. This is obvious from Volcano plots generated for proteins (total peak areas of all corresponding tryptic peptides) detected after FASP without or with prior precipitation of milk and plasma proteins (Figure 4A,B). Although this advantage was more pronounced for plasma, the increase was less than 2-fold for most proteins. Interestingly, the performance of FASP and ISD after protein precipitation depended on the biological sample. While both methods yielded very similar quantitative data for milk (Figure 4C), FASP appeared to be advantageous for plasma (Figure 4D), as most proteins showed more than 2-fold larger peak areas after FASP.

**Figure 5:**
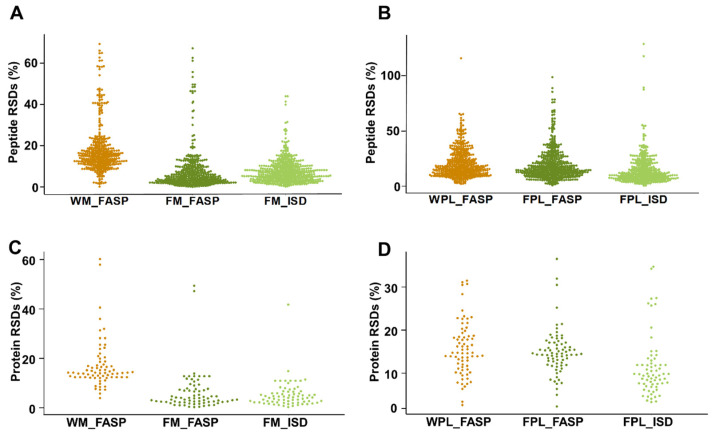
Relative standard deviations (RSDs) for quantifiable peptides in (**A**) human milk and (**B**) plasma, as well as RSDs for the proteins calculated based on the peptide peak area sum for the different sample preparation methods applied to (**C**) human milk and (**D**) plasma. Experiments were performed in three independent replicates.

When quantitation reproducibility was assessed at the peptide and protein levels, precipitation of milk proteins appeared to be favored despite the additional sample preparation steps (Figure 5). Peptides obtained by FM_FASP and FM_ISD could be quantitated with a median RSD% of 6% and 8%, respectively (Figure 5A), while it was only 19% for FASP itself (WM_FASP). This clearly indicates that removal of interfering compounds prior to tryptic digestion is a crucial step in human milk bottom-up proteomics. Unexpectedly, the difference was less pronounced for plasma, as both FASP strategies, i.e., WPL_FASP versus FPL_FASP, showed a similar reproducibility outperforming ISD (Figure 5B). The same trends were observed at the milk and plasma protein levels (Figure 5C,D). The higher RSDs typically derived from low abundant peptides and proteins. When the peak areas of all peptides were correlated between replicates one and two of each method and for both sample types, a very strong correlation confirmed the very reproducible quantitation (Figure S5). The coefficient of determination R2 ranged from 0.988 (WM_FASP) to 0.998 (FM_ISD) in milk and from 0.975 (FPl_FASP) to 0.983 (FPl_ISD) in plasma (Figure S5). 

Interestingly, the peak area sum of spiked DnaK was always higher in milk and plasma samples digested after protein precipitation, independent of FASP and ISD, and the reproducibility of quantitation was better (Figure S6). Both digestion strategies relying on protein precipitation showed similar DnaK quantities (Figure S6), indicating that DnaK it a good internal control to judge analytical strategies in different matrices.

## 3. Discussion

Although a variety of sample preparation methods have been reported for bottom-up proteomics, none can be considered universal for all sample types. The simplest workflows rely on in-solution digestion, despite the drawback of containing all sample-derived contaminations and added reagents that may interfere with proteolysis or LC-MS. Ideally, protein precipitation should precede the ISD followed by desalting prior to LC-MS [11]. Common precipitation methods, using for example TCA, chloroform-methanol, ethyl acetate or acetone, are similarly efficient for a wide variety of samples [12,13,14,15]. However, the Folch procedure (methanol/chloroform/water) was reported to be better for milk proteins [7]. Alternatively, contaminations and reagents can be removed during digestion using ultrafiltration, such as FASP [16]. The current study compared the combination of ISD with preceding protein precipitation and following SPE to FASP with or without preceding protein precipitation. FASP without a prior protein precipitation was the least time consuming, whereas the other two strategies required similar times. Despite possible sample losses during protein precipitation and additionally in SPE after ISD, both FASP and ISD allowed a slightly better protein quantitation in milk and more importantly a higher reproducibility than FASP alone. The latter method might be limited by substances present in biological samples that may interfere with digestion or LC-MS. However, FASP performed better than ISD on plasma protein extracts, but with a lower reproducibility. Thus, FASP of precipitated proteins appears to be the best method for plasma.

The differences among the tested methods were more evident at the peptide level, both in qualitative and quantitative terms. ISD identified more peptides in both milk and plasma, but mainly because it produced many peptides containing missed cleavage sites. This might be a result of the interference of sample components and digestion reagents with the proteolysis step [17]. Previous studies on tissues and cell samples have also reported lower missed cleavage rates for FASP [18,19,20,21]. Interestingly, the missed cleavage rates were lower for milk than for plasma.

Importantly, all methods covered similar parts of the proteomes, typically providing similar sequence coverages for the proteins allowing a reliable profiling of both milk and plasma. Interestingly, milk proteins contained always similar degrees of Cys(CM) and Met(Ox) residues, whereas ISD lead to inefficient carbamidomethylation of plasma proteins, unlike the FASP procedures. A recent study showed lower carbamidomethylation rates for FASP on otolaryngeal tissues than for ISD, but the authors did not perform reduction prior to alkylation [17]. 

Protein precipitation followed by FASP yielded for both sample types higher peptide peaks areas and for milk also a better quantitation reproducibility than FASP alone. Moreover, FASP produced less missed cleavage sites than ISD and thus appears especially advantageous for targeted proteomics, where complete and reproducible proteolysis is crucial for precise quantitation [22].

The spiked DnaK did not affect the digestibility of the sample proteins, nor did the biological matrix affect digestion of DnaK. Therefore, it appears to be a suitable internal digestion control for complex protein mixtures, such as milk and plasma, which should be combined with a mixture of isotopologuos peptides after digestion [23]. 

Due to the high dynamic range of milk and plasma protein concentrations, studies aiming for in-depth proteome characterization should consider additional processing steps, such as depletion or fractionation. However, further sample preparation steps most likely introduce uncertainty, variance, or bias into the data [24]. Therefore, the simplified sample preparation strategies evaluated here are predominantly relevant for large cohort proteomics studies. In particular, when aiming for a correlation of milk and plasma proteomics data, FASP after protein precipitation should be the method of choice. This study was limited to one protein quantity (50 µg), which may not reflect the digestion efficiency for lower or higher protein amounts. The ISD procedure could be further optimized, for example by using two proteases pair (e.g., LysC and trypsin) to improve the cleavage rate for ISD or increasing the solvent volumes to decrease the concentration of interfering substances. However, the latter approach may also negatively affect the digestion efficiency as the protein/trypsin concentration would decrease as well. These aspects should be addressed in future studies in order to optimize the selected sample preparation procedure to its best performance rate. 

## 4. Materials and Methods

Materials were obtained from the following suppliers: AppliChem GmbH (Darmstadt Germany): Iodoacetamide (IAA, ≥99%) and Tris (≥99.9%). Biosolve GmbH (Valkenswaard, Netherlands): Acetonitrile (ULC-MS grade, ≥99.97%), formic acid (ULC-MS grade, ≥99%), and methanol (ULC-MS grade, ≥99.98%). Carl Roth GmbH (Karlsruhe, Germany): Methanol (HPLC grade, ≥99.9%), urea (≥99.5% p.a.), sodium dodecyl sulfate (SDS, ≥99.5%), glycerol (≥99.5%), and dithiothreitol (DTT, ≥99%). Promega GmbH (Mannheim, Germany): sequencing grade modified trypsin. Merck KGaA (Darmstadt, Germany): Chloroform (≥99.8%). Riedel-de Haën (Steinheim, Germany): Bromophenol blue sodium salt. SERVA Electrophoresis GmbH (Heidelberg, Germany): Bovine serum albumin (BSA, >98%), ammonium persulfate (>99%), acrylamide/bis solution (30% *w*/*v*), tetramethylene diamine (≥98.5%), and Coomassie Brilliant Blue G 250. Sigma-Aldrich Chemie GmbH (Steinheim, Germany): Thiourea (≥99%), ammonium bicarbonate (≥99.5%), β-mercaptoethanol (≥99%), sodium deoxycholate (≥97%), and tris-(2-carboxyethyl) phosphine (TCEP, ≥98%). 

Water was purified in-house (resistance >18 mΩ/cm; total organic content < 10 ppb) on a PureLab Ultra Analytic System (ELGA Lab Water, Celle, Germany).

### 4.1. Samples

Experiments with one human milk and one plasma sample, collected from two healthy donors, were conducted in accordance with the Declaration of Helsinki according to a protocol approved by the Ethics Review Board of the Medical Faculty, Leipzig University (277/19-ek for milk and 313/14-ek for blood), with written informed consent from both donors. The samples were stored in aliquots at -80°C and thawed only once prior to analysis. 

### 4.2. Protein Precipitation and Tryptic Digestion

Proteins were precipitated from three aliquots of each milk and plasma sample using Folch extraction [7]. Briefly, milk and plasma samples (50 µL), methanol (375 µL), and chloroform (750 µL) were mixed, incubated under gentle shaking (1 h, 4 °C, 40 rpm), water (625 µL) added, and further incubated (10 min, 4 °C, 40 rpm). The organic phase was removed after centrifugation (10 min, 10,000× *g*, 4 °C), and the remaining sample was centrifuged again to remove the aqueous phase. The protein pellets were immediately dried under vacuum, dissolved in lysis buffer (50 mmol/L Tris-HCl, pH 7.5, 7 mol/L urea, and 2 mol/L thiourea), and the proteins quantified by a Bradford assay relative to a dilution series of bovine serum albumin. Additionally, plasma proteins (dissolved in 50 mmol/L ammonium bicarbonate solution) were quantified on a NanoPhotometer NP80 (IMPLEN, Munich, Germany, λ= 280 nm). Protein contents were further confirmed by SDS-PAGE (T = 15%) using a Coomassie stain [7]. 

Recombinant DnaK, a heat shock protein present in Escherichia coli but not in humans, was expressed and purified in-house [25] and spiked to samples as internal standard. Thus, the standard, as well as human milk and plasma (50 µg protein each) were digested in solution or by FASP without or with DnaK spike (0.25 µg). The samples were spiked with DnaK before digestion (Figure 1). Each sample was processed in triplicates.

Tryptic in-solution digest: Samples (50 µg protein) were diluted with ammonium bicarbonate (25 mmol/L) to obtain a protein concentration of 0.5 g/L. Sodium deoxycholate (1%, *w*/*v*) was added to denature the proteins and TCEP (5 mmol/L) to reduce disulfides (60 °C, 30 min, 550 rpm). Thiols were alkylated with iodoacetamide (10 mmol/L, 37 °C, 30 min, darkness, 550 rpm) and remaining iodoacetamide was quenched with DTT (10 mmol/L, 37 °C, 30 min, 550 rpm). Trypsin was added (1:25 enzyme to protein ratio, 37 °C, overnight, 550 rpm) and the digest stopped with formic acid (0.5%, *v*/*v*). Precipitated sodium deoxycholate was removed by centrifugation (10 min, 9700× *g*) and the supernatant desalted by SPE (Oasis HLB 1 cc, 10 mg, Waters GmbH, Eschborn, Germany) [26]. Briefly, cartridges were washed with methanol and equilibrated with aqueous formic acid (0.1%, *v*/*v*) (1 mL, 2×) before the sample was loaded. Non-binding substances were washed out with aqueous formic acid (0.1%, *v*/*v*) (1 mL, 3×) and the peptides were eluted using aqueous acetonitrile (70%, *v*/*v*) containing formic acid (0.1%, *v*/*v*; 500 µL) and dried under vacuum. 

FASP: The ultrafiltration units (Microcon^®^-10 kDa regenerated cellulose centrifugal filters) were conditioned twice with urea solution (8 mol/L urea in 0.1 mol/L Tris-HCl, pH 8.5, 200 µL, 15 min, 14,000× *g*, 25 °C). Aliquots (50 µg protein) of Folch extracts, whole milk, and plasma with and without spiked DnaK, were diluted with lysis buffer to obtain protein concentrations of 0.5 g/L. Protein samples were reduced with DTT (12.5 µL, 0.5 mol/L in urea solution, 1 h, 37 °C, 550 rpm). Samples were transferred to ultrafiltration units and centrifuged (30 min, 14,000× *g*, 25 °C). Thiols were alkylated with IAA (100 µL, 50 mmol/L in urea solution, 20 min, darkness, RT) and centrifuged (30 min, 14,000× *g*, 25 °C). All samples were washed twice with urea solution (100 µL) and centrifuged (15 min, 14,000× *g*, 25 °C). Ammonium bicarbonate solution (50 mmol/L, 2 × 100 µL) was added and samples were centrifuged (15 min, 14,000× *g*, 25 °C). Proteins were digested with trypsin (1:25 enzyme to protein ratio, 37 °C, wet chamber, overnight). Peptides were transferred into collection tubes via centrifugation (10 min, 14,000× *g*, 25 °C) and filters were washed three times with ammonium bicarbonate solution (50 mmol/L; 2 × 50 µL, 1 × 100 µL; 15 min, 14,000× *g*, 25 °C). Samples were dried under vacuum and reconstituted in aqueous acetonitrile (3%, *v*/*v*) containing formic acid (0.1%, *v*/*v*; 100 µL).

### 4.3. Protein Analysis

Tryptic digests were separated on a nanoACQUITY UPLC (Waters GmbH, Eschborn, Germany) coupled on-line to a Synapt G2-Si mass spectrometer equipped with a nano-ESI source (Waters GmbH, Eschborn, Germany). Peptides (175 ng for milk and 35 ng for plasma digests, 10 µL) were trapped (nanoACQUITY Symmetry C18-column, internal diameter (ID) 180 µm, length 2 cm, particle diameter 5 µm) at a flow rate of 5 µL/min (1% eluent B) and separated on a BEH 130 column (C18-phase, ID 75 µm, length 10 cm, particle diameter 1.7 µm; 35 °C) at a flow rate of 0.3 µL/min. Eluents A and B were water and acetonitrile, respectively, containing formic acid (0.1%, *v*/*v*). Peptides were eluted by two linear gradients starting from 3% to 40% eluent B during 89 min and to 85% eluent B within 5 min [27]. Mass spectra were recorded in positive ion mode (data-dependent acquisition, DDA) using previously reported settings [28]. Briefly, the MS scan time was 0.2 s. Fragmentation was performed in the trap cell using a collision energy ramp (25–50 V for DDA). A GluFib solution (*m*/*z* 785,8426, z = 2) was used to record a lock mass. DDA top 5 was performed from *m*/*z* 360 to *m*/*z* 1600, with an MS/MS scan of 0.4 s using a dynamic exclusion window of 45 s (250 mDa).

### 4.4. Data Analysis

Acquired data sets were imported into PEAKS Studio 10.5 (Bioinformatics Solutions, Waterloo, Canada). After a DeNovo procedure considering cysteine carbamidomethylation (Cys(CM); +57,022 Da) and oxidation of methionine to a sulfoxide (Met(Ox); +15,9949 Da) as variable modifications, tandem mass spectra were searched against the Human Swissprot protein database (accessed on 4 April 2019) additionally containing the DnaK sequence and the cRAP contaminants database (https://www.thegpm.org/crap, accessed on 4 April 2019) considering a precursor mass tolerance of 20 ppm and a fragment mass tolerance of 0.08 Da. Peptides with zero to two missed tryptic cleavage sites were considered for further data processing. Data sets were filtered with a 1% false discovery rate (FDR) at the peptide level and results were exported as tables (pepXML and mzXML).

### 4.5. Relative Quantitation

A spectral library was built with Skyline (v 20.1.0.155) using the pepXML and mzXML files without adding additional FDR thresholds. Identifications with poor quality tandem mass spectra were excluded. Extracted ion chromatograms (XICs) were generated using the first three isotopes of each signal with a TOF resolution of 20,000. Isotopes indicating integration interferences were removed when possible. Integration results were manually filtered for signal-to-noise ratios of at least 10 (S/N ≥ 10) before peak areas were exported.

For statistics, average, standard deviation, and relative standard deviation of peak areas were calculated (Excel 2016) for each peptide in each condition based on the triplicate measurements. For volcano plots the fold change was calculated by dividing the Folch precipitated sample (in-solution digestion) or whole sample (FASP digestion) by the Folch precipitated sample (FASP digestion). A two-sided t-test was performed in order to obtain *p*-values (Excel 2016).

## 5. Conclusions

Sample preparation is a very crucial step in (bottom-up) proteomics. Despite many reports on a variety of protein digestion protocols, each biological matrix may need a specific approach. Here, we compared three digestion strategies for human milk and plasma, i.e., FASP as well as ISD and FASP after protein precipitation, which allowed a reproducible profiling and quantitation of proteins. However, FASP yielded less missed cleavage sites and should be preferred for target peptide quantitation. Quantitation by FASP in combination with protein precipitation was more reproducible for milk, whereas protein precipitation followed by ISD provided a better reproducibility for plasma despite lower peak areas and lower carbamidomethylation degrees (42%). Thus, for each biological material the most efficient and reproducible sample preparation method has to be chosen prior to proteomics studies.

## Data Availability

The data presented in this study are available in the article and the Supplementary Material. The raw data are available on request from the corresponding authors.

## Supplementary Materials

The following are available online, Figure S1: Numbers of DnaK peptides identified in tryptic digests prepared by filter-aided sample preparation (FASP) and in-solution digestion (ISD), the corresponding sequence coverage and numbers of peptides without and with missed cleavage sites. Figure S2: Relative quantities of DnaK peptides without and with one missed cleavage site. Figure S3: Summed peak area of all quantifiable DnaK peptides in digests prepared by FASP and ISD. Figure S4: Overlap of peptides without missed cleavages identified in milk and plasma. Figure S5: Correlation of peptide peak areas for replicates 1 and 2 for each condition in milk and plasma. Figure S6: Summed peak area of spiked DnaK in human milk and plasma. Table S1: DnaK peptides identified by nRPC-ESI-MS/MS in FASP and in-solution digests. Table S2: Peptides identified in FASP and in-solution digests of human milk. Table S3: Peptides identified in FASP and in-solution digests of human plasma.

## Sample Availability

Not available.
