# Supplementary material for: Evaluation of Sample Preparation Strategies for Human Milk and Plasma Proteomics
Source: Molecules. 2021 Nov 11;26(22):6816. doi: 10.3390/molecules26226816 (PMC8618985; doi:10.3390/molecules26226816)
Supplement: Supplementary file 1 [file molecules-26-06816-s001.zip › SupplementFigures.pdf]

Supplementary figures

Evaluation of sample preparation strategies for human milk and plasma proteomics

Sanja Milkovska-Stamenova[a,b*], Michele Wölk[a,b], and Ralf Hoffmann[a,b]

[a]Institute of Bioanalytical Chemistry, Faculty of Chemistry and Mineralogy, Universität Leipzig, Deutscher Platz 5, 04103, Leipzig, Germany

[b]Center for Biotechnology and Biomedicine, Universität Leipzig, Deutscher Platz 5, 04103, Leipzig, Germany

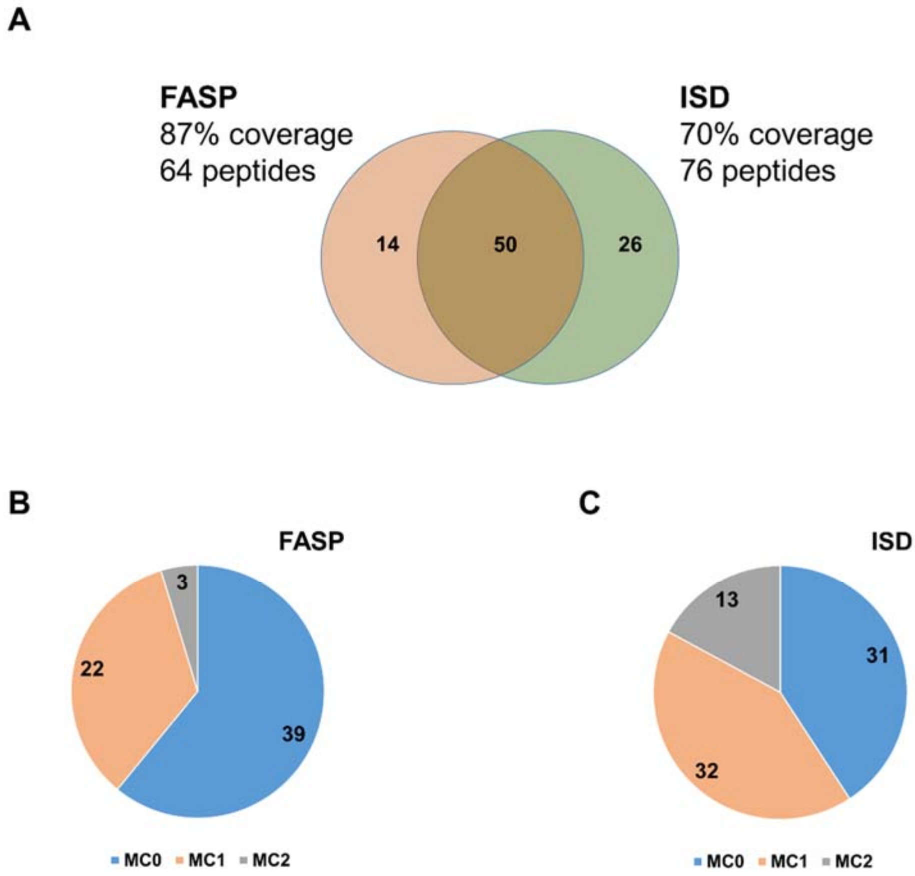

Figure S1: Numbers of DnaK peptides identified in tryptic digests prepared by filter-aided sample preparation (FASP) and in-solution digestion (ISD), the corresponding sequence coverage (A) and numbers of peptides without, with one, and with two missed cleavage sites (pie diagrams, B-C).

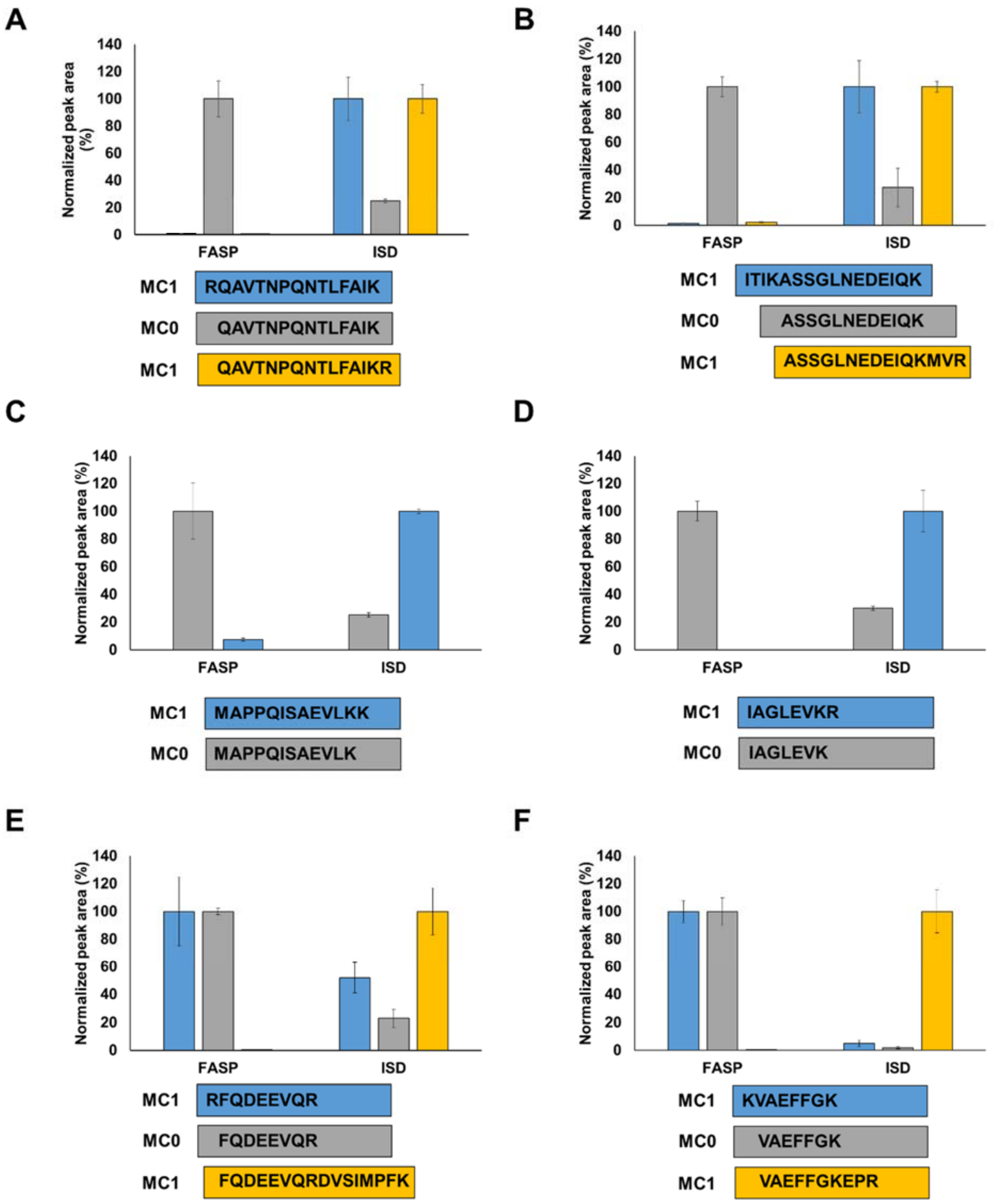

Figure S2: Relative quantities of DnaK peptides without (grey bars) and with one (blue and yellow bars) missed cleavage site. Peak areas are normalized to the highest observed peak area (100 %) for each peptide.

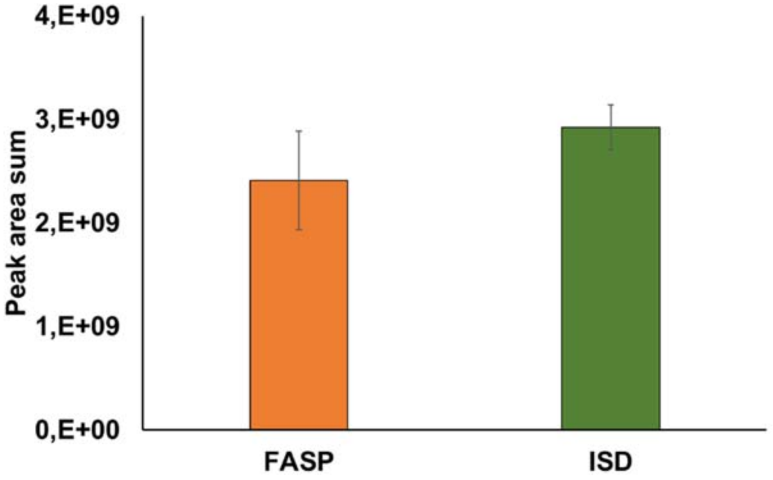

Figure S3: Summed peak area of all quantifiable DnaK peptides in digests prepared by FASP and ISD.

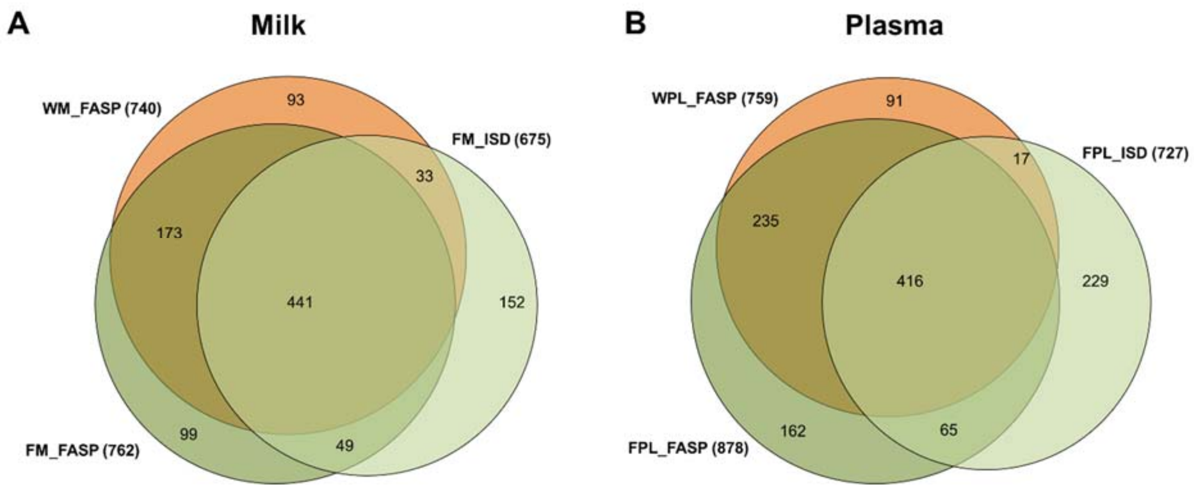

Figure S4: Overlap of peptides without missed cleavages identified in milk (A) and plasma (B)

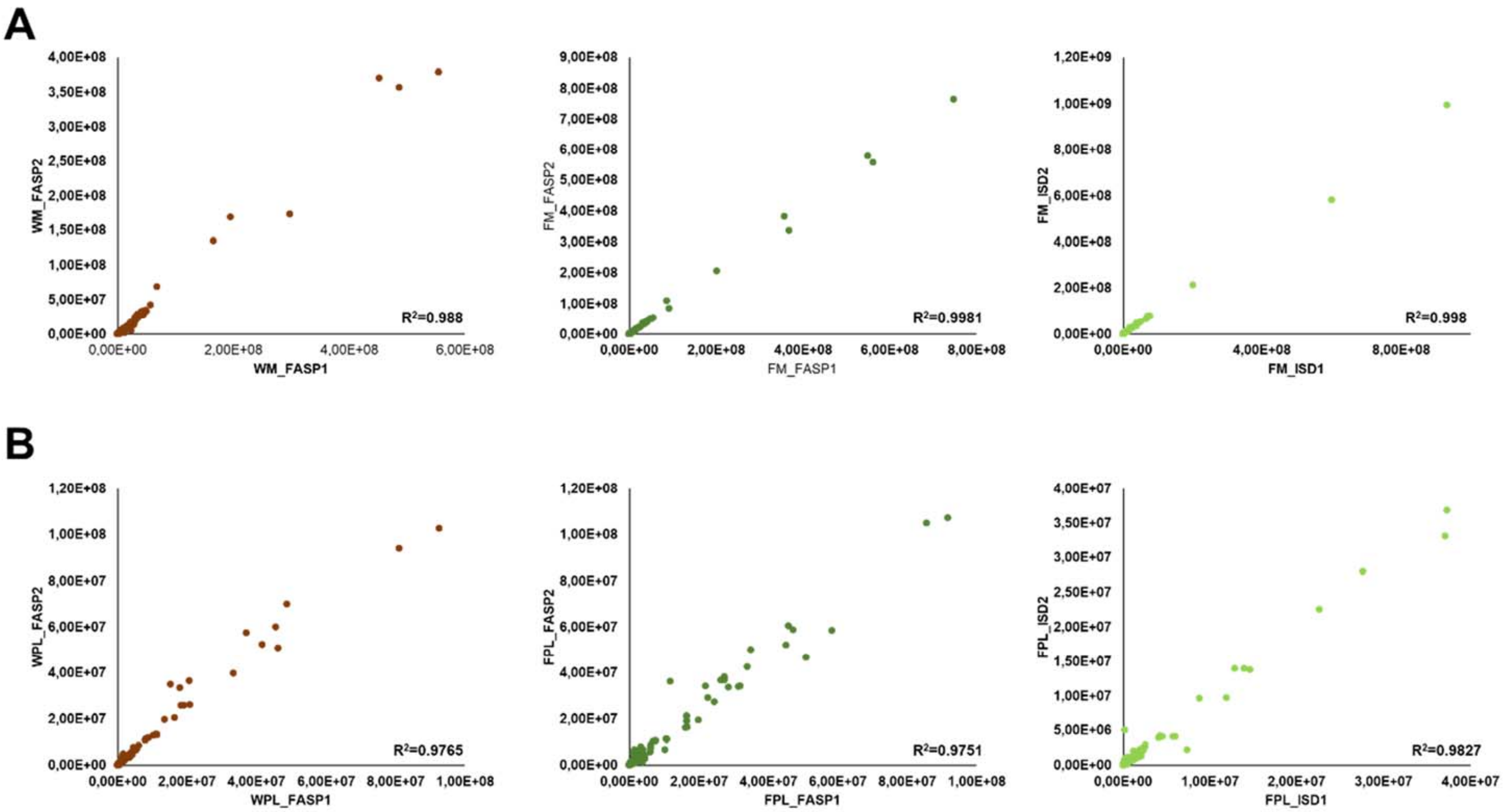

Figure S5: Correlation of peptide peak areas for replicates 1 and 2 for each condition in milk (A) and plasma (B).

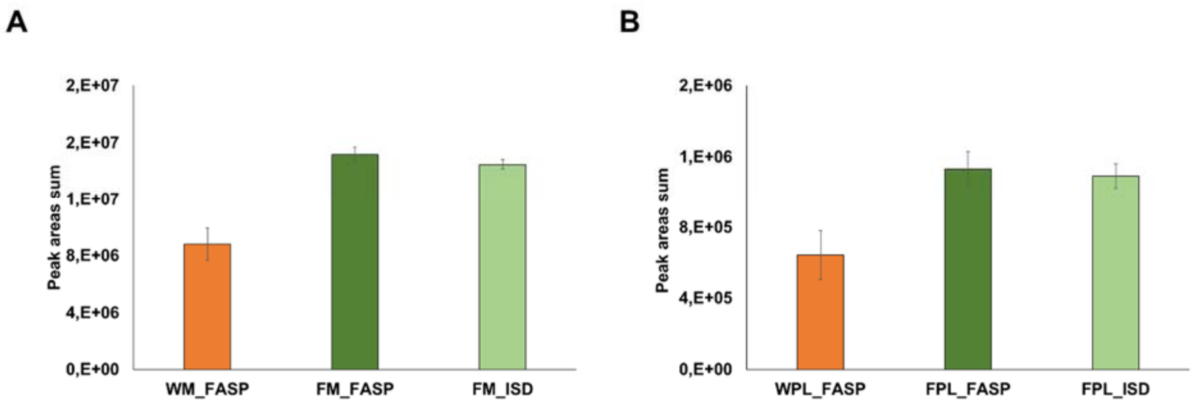

Figure S6: Summed peak area of spiked DnaK in human milk (A) and plasma (B).
